# Fasting plasma mannose levels are associated with insulin sensitivity independent of BMI in Japanese individuals with diabetes

**DOI:** 10.1186/s13098-018-0391-9

**Published:** 2018-12-04

**Authors:** Eri Amano, Shogo Funakoshi, Kumiko Yoshimura, Seiki Hirano, Satoko Ohmi, Hiroshi Takata, Yoshio Terada, Shimpei Fujimoto

**Affiliations:** Department of Endocrinology, Metabolism, and Nephrology, Kochi Medical School, Kochi University, Kohasu, Oko-cho, Nankoku, Kochi 783-8505 Japan

**Keywords:** Insulin resistance, BMI, Mannose, Glucose intolerance

## Abstract

**Background:**

Recently, an integrated network analysis has revealed dysregulation in the metabolism of mannose, a glucose epimer, in severely obese individuals without diabetes. In addition, fasting plasma mannose levels (M_0_) are associated with insulin resistance independent of BMI. Since the association between mannose and insulin sensitivity (IS) in those with impaired glucose tolerance remains unknown, we aimed to investigate this association in individuals without severe obesity but with varying degrees of glucose tolerance.

**Methods:**

Based on 75 g OGTT data in Japanese individuals without diabetic medication, individuals were classified as having normal glucose tolerance (NGT), impaired glucose metabolism (IGM), or diabetes (DM). In each group, 25 individuals were consecutively recruited [total 75 individuals, age: 65 ± 11 (mean ± SD); BMI: 24.9 ± 3.8 kg/m^2^]. QUICKI and Matsuda index (MI) were calculated as IS indices. M_0_ was assayed using HPLC. Normally-distributed log_e_-transformed (ln-) values were used for MI and leptin.

**Results:**

In the simple regression analysis, ln-MI was negatively correlated with BMI (NGT: r = − 0.639, IGM: r = − 0.466, DM: r = − 0.613) and ln-leptin (NGT: r = − 0.480, IGT: r = − 0.447, DM: r = − 0.593) in all 3 groups. Ln-MI was not significantly correlated with M_0_ in NGT (r = 0.241, P = 0.245) and IGT (r = − 0.296, P = 0.152) groups, it was moderately and negatively correlated in the DM group (r = − 0.626, P < 0.001). Similar results were obtained, when QUICKI was used instead of MI as an index of IS. In multiple regression analysis in the DM group, QUICKI (Q) and ln-MI (M) were independently predicted by BMI (Q: β = − 0.413; M: β = − 0.400) and M_0_ (Q: β = − 0.413, M: β = − 0.426), accounting for 51.2% (P = 0.0004) and 51.2% (P = 0.0004) of the variability, respectively, which was larger than the prediction for BMI alone (Q: 38.4%, M: 37.6%).

**Conclusion:**

Fasting plasma mannose was associated with IS independent of BMI in Japanese individuals with DM.

## Background

Obesity is an important factor in the pathogenesis of insulin resistance and substantially increases the risk of type 2 diabetes [1]. To identify novel metabolic biomarkers of obesity, profiling of omics data on obese and lean individuals and a comparison between them has been performed [2–4]. Recently, cell-specific integrated network analysis has revealed dysregulation in the metabolism of mannose in individuals with severe obesity who do not have significant diabetes [4]. Mannose, an epimer of glucose, is a monosaccharide constituent of glycoproteins and glycolipids. In humans, the mean concentrations of plasma mannose are approximately between 40 and 50 μM [5–7]. Interestingly, fasting plasma mannose levels (M_0_) are higher in the obese subjects compared to those in the lean subjects and are associated with insulin resistance independent of BMI. Moreover, downregulation of mannose phosphorylation occurs in the liver of obese individuals, which may lead to decreased plasma mannose utilization and increase in M_0_ [4]. However, the association between mannose and insulin sensitivity in those with diabetes remains unknown, although insulin resistance plays an important role in the pathogenesis of type 2 diabetes [1] and dysregulation of plasma mannose levels has been reported in type 2 diabetes [6, 7]. This study aimed to investigate the association between mannose and insulin sensitivity in individuals with varying degrees of glucose tolerance.

## Methods

### Participants

We analyzed data derived from 75-g oral glucose tolerance test (OGTT) involving 75 Japanese individuals without diabetic medication as described previously [sex (M/F): 34/41; age: 65 ± 11 (mean ± SD); BMI: 24.9 ± 3.8 kg/m^2^] [7].

### Laboratory examinations and anthropometric data

Based on 75 g OGTT data, individuals were either classified as normal glucose tolerance (NGT), impaired glucose metabolism (IGM), or diabetes (DM) according to 2006 WHO criteria [8] [NGT, fasting plasma glucose (FPG) < 6.1 mmol/L and 2-h plasma glucose (2-h PG) < 7.8 mmol/L; IGM, either impaired fasting glucose (IFG, FPG ≥ 6.1 and < 7.0 mmol/L) and/or impaired glucose tolerance (IGT, 2-h PG ≥ 7.8 and < 11.1 mmol/L); DM, FPG ≥ 7.0 mmol/L and/or 2-h PG ≥ 11.1 mmol/L]. In each group, 25 participants were consecutively recruited. Plasma glucose, immunoreactive insulin (IRI), and mannose were measured using the glucose oxidase method, ELISA, and HPLC, as previously described [7]. Serum leptin, a biomarker for body fat mass [9], was measured using ELISA (Quantikine^®^ ELISA, R&D Systems, Minneapolis, MN, USA). Although fasting plasma glucose (G_0_), plasma glucose and mannose 120 min after glucose load (G_120_ and M_120_, respectively), fasting plasma mannose (M_0_)-M_120_, and HbA1c were significantly different among the 3 groups, age, sex, BMI, and M_0_ were not significantly different [7].

### Indices of insulin secretion and insulin sensitivity

Indices of insulin secretion and insulin sensitivity were calculated using glucose and IRI data from the 75-g OGTT, as previously described [7, 10]. QUICKI and Matsuda index (MI) were calculated as indices of insulin sensitivity. The insulinogenic index (IGI) and HOMA-β were calculated as indices of insulin secretion. The oral disposition index (DI_O_) was calculated as an index of insulin secretion adjusted for insulin sensitivity. IGI, QUICKI, MI and DI_O_ were significantly different among the NGT, IGM, and DM groups, but HOMA-β was not significantly different [7].

### Statistical analysis

Non-normally distributed continuous data are presented as median values, and 25th and 75th percentile values. The Kruskal–Wallis test was used to determine differences for non-normally distributed continuous data among more than three groups. The relationship between the parametric data was determined using Pearson analysis. In regression analyses, Normally-distributed log_e_-transformed (ln-) values were used for MI, leptin, and high-density lipoprotein-cholesterol (HDL-C). P values < 0.05 were considered statistically significant.

## Results

### Simple regression analyses

**Fig. 1:**
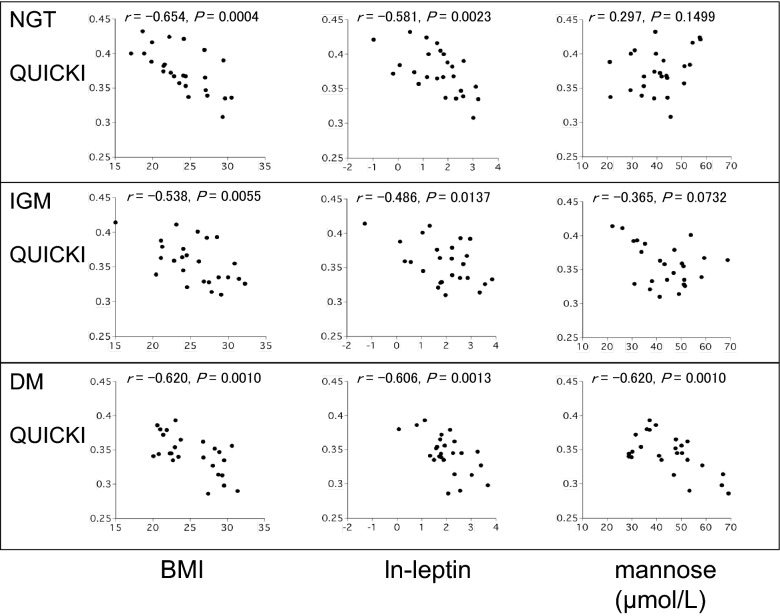
Relationships between QUICKI and clinical factors including BMI, ln-leptin, and fasting plasma mannose in NGT, IGM and DM groups (*n* = 25 in each group)

**Fig. 2:**
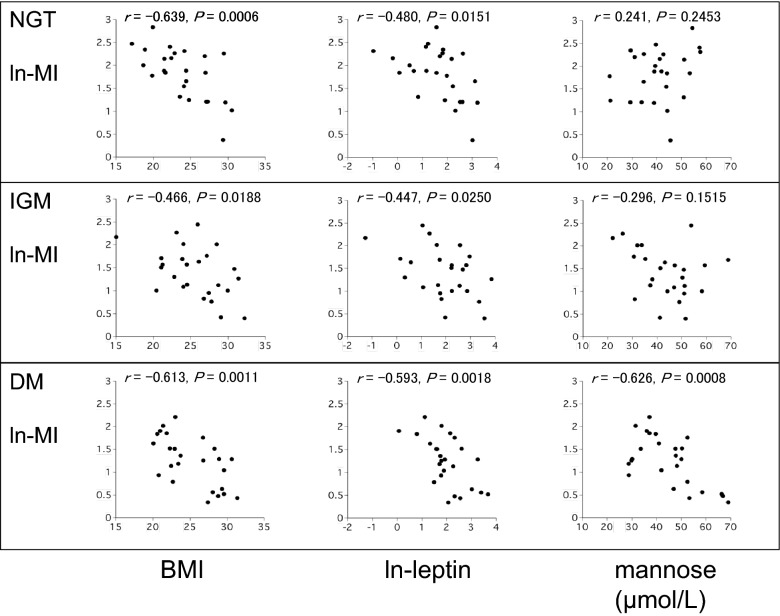
Relationships between ln-MI and clinical factors including BMI, ln-leptin, and fasting plasma mannose in NGT, IGM and DM groups (*n* = 25 in each group)

Serum leptin and HDL-C were not significantly different among the 3 groups (Table 1). QUICKI was negatively correlated with BMI (NGT: r = − 0.654, IGM: r = − 0.538, DM: r = − 0.620) and ln-leptin (NGT: r = − 0.581, IGM: r = − 0.486, DM: r = − 0.606) in all 3 groups. Ln-MI was negatively correlated with BMI (NGT: r = − 0.639, IGM: r = − 0.466, DM: r = − 0.613) and ln-leptin (NGT: r = − 0.480, IGM: r = − 0.447, DM: r = − 0.593) in all 3 groups (Table 2, Figs. 1, 2). Interestingly, although QUICKI and ln-MI were not significantly correlated with M_0_ in the NGT group (QUICKI: r = 0.297, *P *= 0.1499; ln-MI: r = 0.241, *P *= 0.2453) and in the IGM group (QUICKI: r = − 0.365, *P *= 0.0732; ln-MI: r = − 0.296, *P *= 0.1515), they were moderately and negatively correlated in the DM group (QUICKI: r = − 0.620, *P *= 0.0010; ln-MI: r = − 0.626, *P *= 0.0008) (Table 3, Figs. 1, 2). In contrast, a decrease in mannose levels after a glucose load (M_120_ − M_0_) was associated with QUICKI in the NGT group (r = − 0.462, P = 0.0210) but not in the IGM and DM groups (IGM: r = 0.295, *P *= 0.1571; DM: r = 0.066, *P *= 0.7538) (Table 3).

**Table 1:** Serum leptin and HDL-C levels in the NGT, IGM and DM groups

	NGT	IGM	DM	*P* value
n	25	25	25	
Leptin (ng/mL)	6.08 (2.28,12.35)	7.17 (2.92,16.88)	6.57 (4.53,12.79)	0.5011
HDL-C (mg/dL)	59.6 (50.2,65.1)	50.2 (43.8,62.0)	55.2 (48.0,64.5)	0.0841

Median values are shown. The 25th percentile and 75th percentile values are shown in parenthesis

**Table 2:** Simple correlation between insulin sensitivity indices and clinical factors in the NGT, IGM, and DM groups

	QUICKI					
	NGT		IGM		DM	
	r	*P*	r	*P*	r	*P*
Age	0.146	0.4827	0.611	0.0012	0.600	0.0015
Sex	0.197	0.3443	− 0.117	0.5789	− 0.255	0.2189
BMI	− 0.654	0.0004	− 0.538	0.0055	− 0.620	0.0010
WC	− 0.600	0.0015	− 0.399	0.0483	− 0.470	0.0178
HbA1c	− 0.342	0.0943	0.179	0.3931	− 0.354	0.0827
Log_e_-leptin	− 0.581	0.0023	− 0.486	0.0137	− 0.606	0.0013
Log_e_-HDL-C	0.272	0.1879	− 0.022	0.9176	0.273	0.1875

	Log_e_-MI					
	NGT		IGM		DM	
	r	*P*	r	*P*	r	*P*
Age	0.103	0.6257	0.614	0.0011	0.527	0.0068
Sex	0.046	0.8257	− 0.116	0.5815	− 0.260	0.2096
BMI	− 0.639	0.0006	− 0.466	0.0188	− 0.613	0.0011
WC	− 0.573	0.0027	− 0.419	0.0369	− 0.535	0.0059
HbA1c	− 0.289	0.1610	0.165	0.4312	− 0.215	0.3026
Log_e_-leptin	− 0.480	0.0151	− 0.447	0.0250	− 0.593	0.0018
Log_e_-HDL-C	0.128	0.5415	− 0.054	0.7962	0.231	0.2657

*G*_*0*_ fasting plasma glucose, *WC* waist circumference, *MI* Matsuda Index

**Table 3:** Simple correlation between insulin sensitivity/secretion indices and plasma mannose levels in the NGT, IGM, and DM groups

	M_0_					
	NGT		IGM		DM	
	r	*P*	r	*P*	r	*P*
Log_e_-IGI	− 0.177	0.5768	− 0.056	0.7913	0.381	0.0599
Log_e_-HOMAβ	− 0.149	0.4781	0.023	0.9115	0.252	0.2234
Log_e_-DI_O_	0.068	0.7458	− 0.225	0.2795	− 0.057	0.7852
QUICKI	0.297	0.1499	− 0.365	0.0732	− 0.620	0.0010
Log_e_-MI	0.241	0.2453	− 0.296	0.1515	− 0.626	0.0008

	M_120 _− M_0_					
	NGT		IGM		DM	
	r	*P*	r	*P*	r	*P*
Log_e_-IGI	0.303	0.1407	− 0.249	0.2305	− 0.323	0.1150
Log_e_-HOMAβ	0.330	0.1067	− 0.141	0.5026	− 0.177	0.3917
Log_e_-DI_O_	0.007	0.9741	− 0.078	0.7105	− 0.325	0.1130
QUICKI	− 0.462	0.0201	0.295	0.1571	0.066	0.7538
Log_e_-MI	− 0.384	0.0578	0.273	0.1868	0.202	0.3325

M_0_ and M_120_ are plasma mannose levels at 0 (fasting) and 120 min after glucose loading. M_120 _− M_0_ is alteration of mannose levels during glucose loading (M_120_ minus M_0_)

*IGI* insulinogenic index, *DI*_*O*_ oral disposition index, *MI* Matsuda index

### Multiple regression analyses in the DM group

QUICKI was independently predicted by BMI (β = − 0.413) and M_0_ (β = − 0.413), accounting for 51.2% (*P *= 0.0004) of the variability (Model 1), and by ln-leptin (β = − 0.414) and M_0_ (β = − 0.438) accounting for 52.2% (*P *= 0.0003) of the variability (Model 2) (Table 4), which was larger than that for predictions by BMI alone (38.4%) and ln-leptin alone (36.7%). Ln-MI was independently predicted by BMI (β = − 0.400) and M_0_ (β = − 0.426), accounting for 51.2% (*P *= 0.0004) of the variability (Model 3), and by ln-leptin (β = − 0.394) and M_0_ (β = − 0.453) accounting for 51.7% (*P *= 0.0003) of the variability (Model 4) (Table 4), which was larger for predictions by BMI alone (37.6%) and ln-leptin alone (35.1%).

**Table 4:** Multiple regression analyses for the determinants of insulin sensitivity in the DM group

Dependent variable	Independent variables	Std. coef.	R^2^
QUICKI	BMI	− 0.413 (0.0253)	0.512 (0.0004)
(Model 1)	M_0_	− 0.413 (0.0253)	
QUICKI	Log_e_-leptin	− 0.414 (0.0193)	0.522 (0.0003)
(Model 2)	M_0_	− 0.438 (0.0140)	
Log_e_-MI	BMI	− 0.400 (0.0298)	0.512 (0.0004)
(Model 3)	M_0_	− 0.426 (0.0215)	
Log_e_-MI	Log_e_-leptin	− 0.394 (0.0258)	0.517 (0.0003)
(Model 4)	M_0_	− 0.453 (0.0118)	

M_0_ and G_0_ are fasting plasma mannose and glucose levels, respectively

Probabilities are indicated in parentheses

*MI* Matsuda index

## Discussion

Insulin resistance is established as a precursor of type 2 diabetes and cardiovascular disease [1]. Since insulin resistance is commonly found in obesity and weight reduction improves insulin resistance, adiposity plays an important role in the pathogenesis of insulin resistance. Although the mechanism by which obesity causes insulin resistance has not been fully elucidated, systemic inflammation, adipose-derived proinflammatory molecules, and free fatty acids may play important roles in the increase in insulin resistance [1]. However, obesity alone cannot explain insulin resistance completely. On the basis of this background, exploratory studies to find novel biomarkers related to insulin resistance independent of adiposity have been performed that have compared the metabolic profile between obese individuals without significant diabetes and lean individuals using metabolomics. These studies have revealed that branched-chain amino acids including leucine, isoleucine, and valine [2] and α-hydroxybutyrate [3] are associated with insulin sensitivity independent of adiposity. Recently, a cell-specific integrated network analysis that merged genome-scale metabolic, transcriptional regulatory, and protein–protein interaction networks revealed dysregulation in the metabolism of mannose in individuals with severe obesity who did not have significant diabetes [4]. These results showed that the expression level of hexokinase, which mediates a limited step reaction in mannose utilization in the liver, was reduced in obese individuals. In addition, insulin sensitivity was associated with the fasting plasma mannose level independent of BMI. In the present study, an association between fasting plasma mannose levels and insulin sensitivity independent of BMI was observed in individuals with diabetes and not in individuals without diabetes. These results suggest the contribution of factors other than adiposity towards insulin resistance in diabetes. Prediction of insulin sensitivity by BMI and fasting plasma mannose levels was more accurate than the prediction by BMI alone, which may contribute to more precise estimation of insulin sensitivity in patients with diabetes.

Since it has recently been reported that plasma mannose levels are significantly associated with a future risk of type 2 diabetes in cohort studies [11, 12], mannose metabolism might be dysregulated in the early phase of glucose intolerance. Fasting plasma mannose levels of individuals with diabetes (DM) are higher than those of individuals with NGT [6] and there is a close positive correlation between plasma glucose and mannose levels [13–15]. Experiments in rats have previously shown that the plasma mannose level decreases after glucose loading, but does not decrease in diabetic rats, and that hepatic glycogenolysis is a source of this plasma mannose [16]. We have clarified the relation between plasma mannose level and glucose tolerance in humans [7]. After glucose loading, the plasma mannose level decreased gradually in the NGT group, but did not decrease in the DM group. The plasma mannose changes, throughout 120 min following oral glucose loading from base line (M_120 _− M_0_), were significantly different among the NGT, IGM, and DM groups and were correlated with indices of insulin secretion and sensitivity. These results suggest that insufficient suppression of glycogenolysis due to impaired insulin secretion after glucose loading and hepatic insulin resistance in those with diabetes is linked to an enhanced mannose efflux from the liver and a blunted decrease in mannose levels after glucose loading in those with diabetes. The alteration of mannose utilization in the liver in diabetes remains unknown, and whether reduced mannose phosphorylation and utilization in the liver also occur in non-obese individuals with diabetes should be elucidated. Our results showing that M_0_ was correlated with QUICKI in the DM group only and that M_120 _− M_0_ was correlated with QUICKI in the NGT group only suggest the influence of different ranges of insulin sensitivity on fasting and post-glucose-load mannose metabolism.

### Limitations

The present study has several limitations. First, insulin sensitivity was not measured using a hyperinsulinemic-euglycemic clamp, which is the gold standard method. Second, since this study was cross-sectional, a causal association could not be evaluated. Third, this study may have been affected due to selection bias and not be representative of the broader population, since data were collected at a single institute in Japan. Further multicenter collaborative research is needed.

## Conclusion

This study established that fasting plasma mannose was correlated with insulin sensitivity independent of BMI in Japanese individuals with diabetes.

## Abbreviations

OGTT: oral glucose tolerance test
IRI: immunoreactive insulin
ELISA: enzyme-linked immunosorbent assay
HbA1c: glycated hemoglobin
WHO: World Health Organization
NGT: normal glucose tolerance
IGM: impaired glucose metabolism
IFG: impaired fasting glucose
G: glucose
I: IRI
IGI: insulinogenic index
HOMA: homeostasis model assessment
QUICKI: quantitative insulin sensitivity check index
DI_O_: oral disposition index
HDL-C: high-density lipoprotein-cholesterol
ANOVA: analysis of variance
BMI: body mass index
